# Male Breast Cancer Review. A Rare Case of Pure DCIS: Imaging Protocol, Radiomics and Management

**DOI:** 10.3390/diagnostics11122199

**Published:** 2021-11-25

**Authors:** Daniele Ugo Tari, Luigi Morelli, Antonella Guida, Fabio Pinto

**Affiliations:** 1 Department of Diagnostic Senology, District 12, Palazzo della Salute, Caserta LHA, 81100 Caserta, Italy; 2 Department of Pathological Anatomy A. di Tuoro, Caserta LHA, 81031 Aversa, Italy; morelliluigi1@gmail.com; 3 Head Office, District 12, Palazzo della Salute, Caserta LHA, 81100 Caserta, Italy; antonella.guida@aslcaserta.it; 4 Department of Radiology, A. Guerriero Hospital, Caserta LHA, 81025 Marcianise, Italy; fpinto1966@libero.it

**Keywords:** male breast cancer, breast neoplasms, clinical practice patterns, ductal carcinoma in situ, radiomics, digital breast tomosynthesis, proliferative disease of the breast, guidelines

## Abstract

Ductal carcinoma in situ (DCIS) of male breast is a rare lesion, often associated with invasive carcinoma. When the in situ component is present in pure form, histological grade is usually low or intermediate. Imaging is difficult as gynaecomastia is often present and can mask underlying findings. We report a rare case of pure high-grade DCIS in a young male patient, with associated intraductal papilloma and atypical ductal hyperplasia. Digital breast tomosynthesis (DBT) showed an area of architectural distortion at the union of outer quadrants of the left breast without gynaecomastia. Triple assessment suggested performing a nipple-sparing mastectomy, which revealed the presence of a focal area of high-grade DCIS of 2 mm. DCIS, even of high grade, is difficult to detect with mammography and even more rare, especially when associated with other proliferative lesions. DBT with 2D synthetic reconstruction is useful as the imaging step of a triple assessment and it should be performed in both symptomatic and asymptomatic high-risk men to differentiate between malignant and benign lesions. We propose a diagnostic model to early detect breast cancer in men, optimizing resources according to efficiency, effectiveness and economy, and look forward to radiomics as a powerful tool to help radiologists.

## 1. Introduction

Male breast cancer (MBC) is a rare entity representing less than 1% of all male malignancies and less than 1% of breast cancers. The annual incidence of MBC in Europe is approximately 1/100,000 men. Ductal carcinoma in situ (DCIS) of the male represents approximately 0.1% of all breast cancers and less than 0.1% of all cancers in men [1,2,3].

The most relevant risk factor for MBC is the increasing age, probably related to testicular malfunction and increased levels of estrogen, so that the age of onset varied between 60 and 70 years with an average age of 67, 5 to 10 years older than women at the time of diagnosis [1,4]. Other risk factors include family history (positive in 15 to 20% of men), mutations of breast cancer related genes (BRCA2 > BRCA1), Cowden and Klinefelter syndromes, alcohol consumption, and liver disease. Although claims have been made to the contrary, there is no proven direct link between gynaecomastia and MBC [1]. Since it is difficult to diagnose MBC at an early stage, it could be useful to define a specified protocol according to the literature and future perspectives. In this paper, we present a rare case of pure high-grade DCIS of the male breast in a young patient. Furthermore, starting from this experience, we reviewed the literature to define the correct use of breast imaging according to the criteria of efficiency, effectiveness and economy, presenting a diagnostic algorithm and also showing the possibilities of radiomics.

## 2. Case Report

A 43-year-old Caucasian male reported a 1-month history of spontaneous clear left side nipple discharge with a recent appearance of a homolateral painless breast swelling. There was no history of bloody discharge. Past medical history was pertinent for obesity class I (BMI: 33.3) and bilateral hypoacusia for otosclerosis. There was no family history for breast or ovarian cancer. His social history indicated no use of alcohol, but previous use (twelve years ago) of tobacco products.

On physical examination, he was an overweight Caucasian male with symmetrical breasts. On palpation, there was a bilateral pseudogynaecomastia with a smooth, ill-defined left breast thickening, especially at the union of the outer quadrants. With applied pressure, a minimal clear stream of discharge fluid was elicited from the left nipple and was felt to be localized to a single duct.

**Figure 1:**
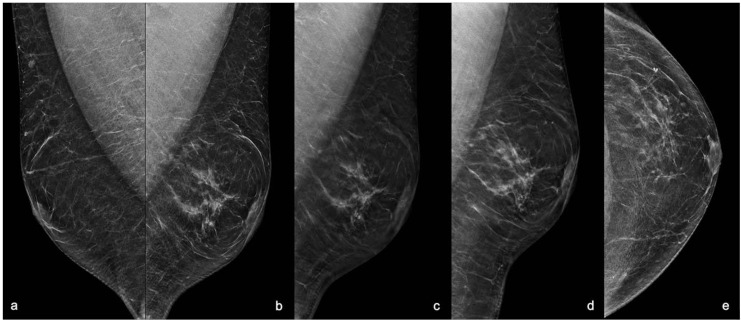
Mammography with s2D and DBT projections. (**a**) Right MLO s2D showing normal appearance of male breast with false gynaecomastia. (**b**) Left MLO s2D; (**c**) Left MLO DBT; (**d**) Left LM s2D; (**e**) Left CC s2D. 2D mammograms showed an area of asymmetric density at the union of outer quadrants of the left breast with regular breast buttons in absence of gynaecomastia. DBT showing the area of architectural distortion at the union of outer quadrants of the left breast with rare punctate microcalcifications and small peripheral pseudo-nodular opacities referred to focal ductal ectasia, sparing the nipple-areolar complex. MLO: medio-lateral-oblique projection; LM: latero-medial projection; CC: cranio-caudal projection; s2D: 2D synthetic reconstruction; DBT: digital breast tomosynthesis.

Digital breast tomosynthesis (DBT) with synthesized reconstructed 2D images (s2D) was performed in medio-lateral-oblique (MLO) projections for each breast and in both cranio-caudal (CC) and latero-medial (LM) projections for the left breast. The s2D images showed a regular appearance of the breast buttons without gynaecomastia, and an area of asymmetrical density at the union of outer quadrants of the left breast that was better identified at the DBT images as an area of architectural distortion with scattered peripheral punctate calcifications, sparing the nipple-areolar complex. (Figure 1).

**Figure 2:**
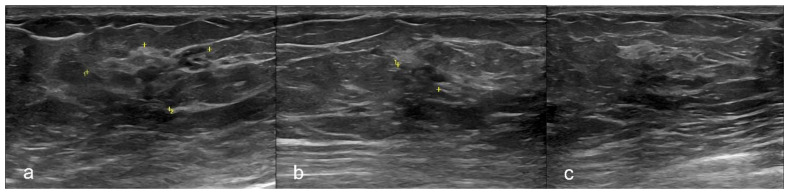
Ultrasonography. Breast ultrasound (US) in different projections showing an ill-defined, hypoechoic area of acoustic shadowing with peripheral anechoic lacunae at the union of outer quadrants of the left breast. (**a**) The yellow mark showed the whole area. (**b**) The yellow mark showed the small area of architectural distortion with acoustic shadowing. (**c**) US-guided Fine-Needle Aspiration Cytology (FNAC) of the lesion.

A breast ultrasound (US), performed on the same day, showed in correspondence of the mammographic findings, the presence of an ill-defined, hypoechoic area of acoustic shadowing with peripheral anechoic lacunae and a close small focal ductal ectasia. (Figure 2)

According to Breast Imaging Reporting and Data System (BI-RADS) [5], these findings were classified as category 4b.

**Figure 3:**
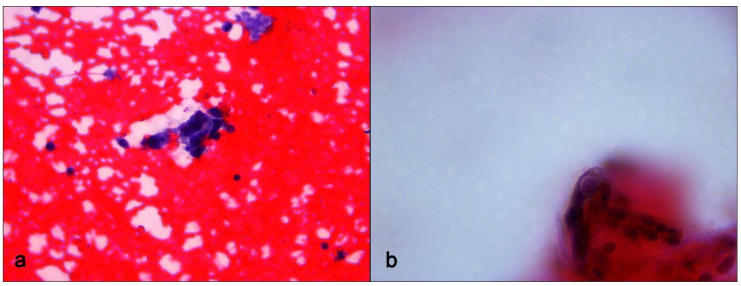
Fine-Needle Aspiration Cytology sampling. (**a**) Small cluster of ductal cells showing loose aggregation, irregular chromatin pattern and nuclear polymorphism. Papanicolaou stain, ×400 magnification. (**b**) Small cluster of ductal cells showing cellular details. Papanicolaou stain, ×600 magnification.

**Figure 4:**
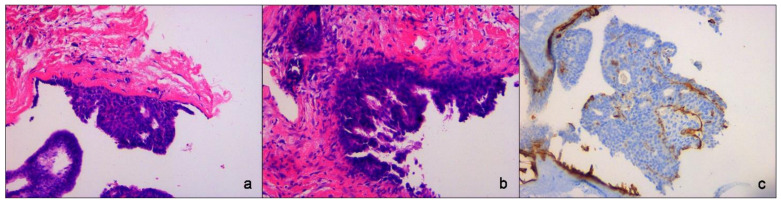
Core-Needle Biopsy sampling. (**a**,**b**) Atypical Ductal Hyperplasia (ADH) with true papillary projections. Hematoxylin eosin stain, ×200 magnification. (**c**) Immunohistochemistry for p63 showing myoepithelial component, ×200 magnification.

An US-guided Fine-Needle Aspiration Cytology (US-FNAC) was performed. Our laboratory received 4 microscope slides fixed in alcohol and coloured with Papanicolaou stain. Microscopic examination showed atypical ductal cells with poor myoepithelium arguing for several types of proliferative non-malignant lesions but also DCIS. According to the IAC Yokohama System for Reporting Breast Fine-Needle Aspiration Biopsy Cytopathology (1st Edition, 2020) [6], these findings were classified as category C4 (Figure 3). Since the Rapid On-Site Evaluation (ROSE) [7] already showed this suspicious atypia, we contextually performed an US-guided biopsy (US-CNB) with a 16 Gauge (G) semi-automated core biopsy needle. Our laboratory received two specimens of breast tissue of 1.5 cm of maximum dimension who were submitted for routine processing. Microscopic examination showed breast parenchyma with fibrosis with a focal area of atypical ductal hyperplasia (ADH), p63 positive and with focal positivity for CK5/6. According to Guidelines for non-operative diagnostic procedures and reporting in breast cancer screening [8], these findings were classified as category B3 (Figure 4).

**Figure 5:**
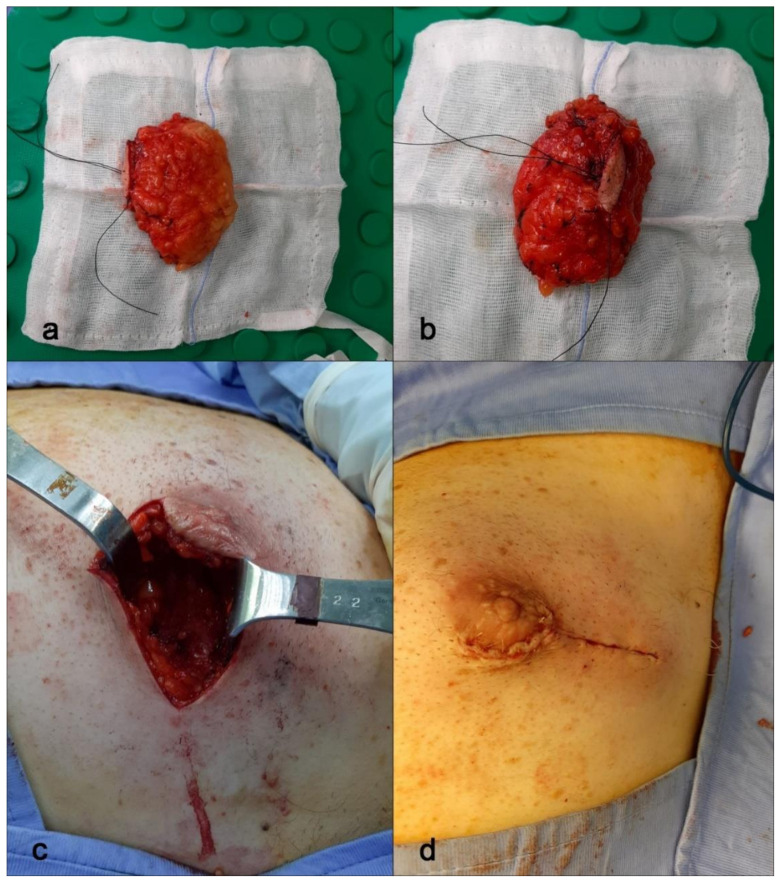
Nipple-sparing mastectomy. (**a**,**b**) Breast tissue samples from mastectomy surgery. Here, the entire breast sample. (**c**,**d**) Periareolar incision with lateral extension and breast reconstruction.

All the findings of cytology and histology in association with the radiological assessment underlined the importance of the triple test [9] and recommended us to perform surgical excision of the suspected area. Therefore, after discussing with the multidisciplinary team (MDT), according to the patient’s consent, a left subcutaneous nipple-sparing mastectomy was performed without sentinel lymph node biopsy (Figure 5).

**Figure 6:**
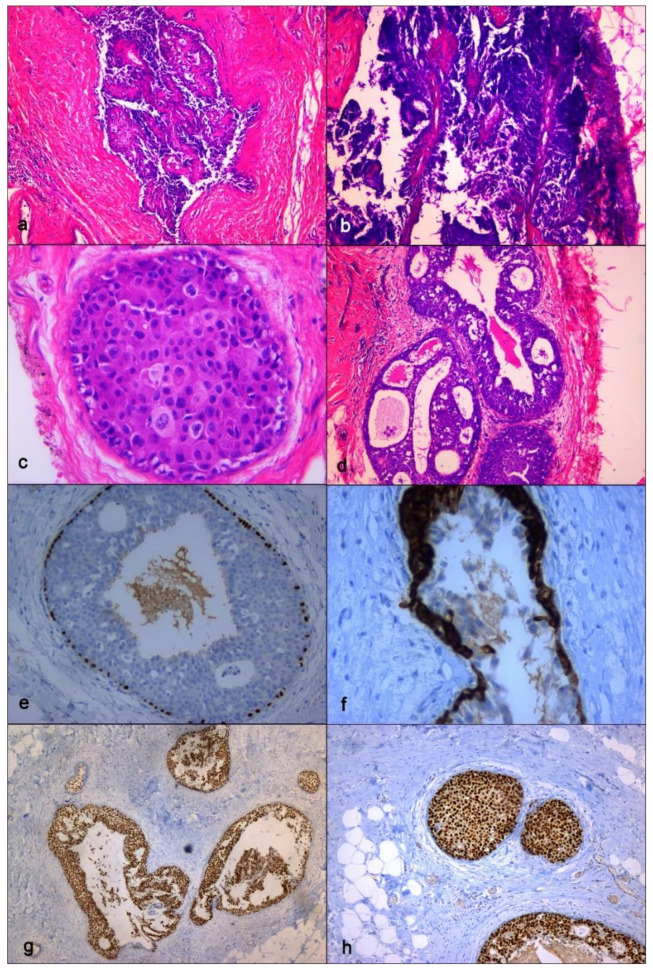
Definitive histopathological specimen. (**a**) Intraductal papillary lesion with epithelial atypia, hematoxylin eosin stain, ×50 magnification; (**b**) Papillary lesion representative of epithelial atypia at higher magnification, hematoxylin eosin stain, x100 magnification; (**c**) High grade ductal carcinoma in situ (DCIS), hematoxylin eosin stain, ×400 magnification; (**d**) High grade DCIS, solid-cribriform type, hematoxylin eosin stain, ×400 magnification; (**e**) Immunohistochemstry (IHC). Study of p63 expression showing immunoreactivity of myoepithelial cell along the basement membrane, ×200 magnification; (**f**) IHC. Study of cytokeratin 5/6 immunoreactivity showing Atypical Ductal Hyperplasia (AD), ×400 magnification; (**g**) IHC. Study of estrogen receptor (ER) immunoreactivity with 70% of nuclei staining with strong intensity, ×50 magnification; (**h**) IHC. Study of progesterone receptor (PR) immunoreactivity with 80% of nuclei staining with strong intensity, ×100 magnification.

Our laboratory received a specimen designated left mastectomy, weighing 90 g (Figure 5). It comprised oriented fibro-fatty tissue, 8 × 4.5 × 4 cm in aggregate, which on gross examination revealed an area of small cystic formations and a whitish lesion of 0.2 cm on which random samplings were performed. Microscopic examination showed an intraductal papilloma (IP) with ADH and a single focus of DCIS solid-cribriform type, respectively. Nuclear grade was 3 (p63+, CK5/6−) with negative surgical margins. No invasive cancer was present (Figure 6). The specimen was sent for immunohistochemical examination of estrogen (ER) and progesterone (PR) receptor. The tissue was positive for both ER (with 70% of nuclei staining with strong intensity) and PR (80% of nuclei staining strongly) (Figure 6). Venipuncture samples were subsequently sent to an external laboratory for genetic analysis, the results of which were negative for BRCA1 and 2, and other mutations (Table 1).

According to these surprising results, after a clinically and radiologically evaluation of the left axillary lymph nodes, the MDT decided not to perform axillary dissection and to administer tamoxifen as approved adjuvant hormone treatment of men with ER-positive early stage breast cancer [10].

The patient was noted to be doing well 6 months post-operatively and is still on follow-up at the time of writing.

## 3. Discussion

If male breast cancer (MBC) is a rare disease, atypical ductal hyperplasia (ADH), intraductal papilloma (IP) and a pure high-grade ductal carcinoma in situ (DCIS), with no associated gynaecomastia and significant risk factors, are even more rare, especially if they occur concomitantly [11,12,13].

Despite its rarity, the incidence of MBC has increased by 20–25% in the past few decades and continues to rise [14]. Nevertheless, clinical research is limited and most available data come from observational retrospective studies [14,15,16,17].

Presenting symptoms are similar to female breast cancer (FBC). Often, MBC presents as a nodule found on self-examination (91.5%). The main presenting symptom is a painless retro-areolar mass (50–95%). Other clinical signs either isolated or in association with the breast mass are mastodynia, breast discharge (1–12%), inflammatory signs, ulceration (4–17%) or symptoms related to a metastatic disease (3–12%) [18]. Nipple abnormalities are early and more frequent than in women (40–50%) because of the low volume of breast tissue in men and the central location of some tumours. Paget’s disease complicates 1.45% of male breast cancers versus 0.68% in women [19].

Unlike women, a definition for men at “high risk” for breast cancer has not yet been established. One review paper considered men at “high risk” if they had a lifetime risk greater than the male average risk [20]. As reported in literature, we can consider in this category all the men with a known BRCA genetic mutation, Ashkenazi Jewish ancestry, a strong family history, or a personal history of breast cancer [20,21].

Several genes have been reported to be mutated, such as BRCA2 (4–16%), BRCA1 (0–4%), PTEN (phosphatase and tensin homolog), p53, CHEK2 (checkpoint kinase 2), PALB2 (partner and localizer of BRCA2) and CYP17A1 (cytochrome P450 family 17 subfamily A member 1) [22]. Therefore, the American Society of Clinical Oncology (ASCO) recommends that genetic counselling and testing regardless of family history should be offered to all men with breast cancer [4]. Additional risk factors include obesity, testicular abnormalities or pituitary adenomas that led to hormonal imbalance (increased level of estrogen), hepatic disease (cirrhosis), hormonal exogenous estrogens therapies, race (black men) and radiation exposure.

In our case, the patient did not show any of the previous described risk factors, except for a class I obesity according to BMI Index. Furthermore, no gynaecomastia was found and even if there is no proven direct link of its influence on MBC [1], it could be considered an important limitation on breast imaging due to its aspects that could mask underlying lesions. In fact, we can identified three patterns of gynaecomastia seen at mammography (MX): nodular, dendritic, and diffuse glandular [23]. While gynaecomastia is central and most commonly bilateral, MBC can occasionally develop in the sub-areolar region, but more often, it tends to spare this region resulting in an eccentric unilateral non-tender mass. Nipple retraction is seen due to the late diagnosis that led also to skin thickening and axillary adenopathy [24].

In our patient, the lesion was found to be an eccentric area of architectural distortion extending to the union of outer quadrants, with no relationship with the nipple-areolar complex. It was an ill-defined tender mass that could be underrated in the absence of other clinical findings as the nipple discharge described by the patient himself. The absence of other clinical relevant findings on palpation is due to the early onset of the pathology and the intraductal development of the cancer that was detected prior to progression to invasion.

The median duration of symptoms for pure DCIS is 2 months, whereas for patients with DCIS and associated invasive carcinoma it is 6 months [25]. In our case, the patient had breast pain for 1-month with nipple discharge but the pathological analysis of breast tissue also showed an area of focal ductal ectasia with a papilloma, near the DCIS, which were probably responsible for these symptoms.

The vast majority of male breast cancers are invasive ductal carcinomas. Invasive lobular carcinoma is rare (1%), presumably due to the lack of lobular development in the male breast [1]. Lobular carcinoma in situ has been reported but usually in association with an invasive lobular carcinoma [26]. If male DCIS represents approximately 10% of all male breast cancers, pure DCIS only accounts for 5% of these cases, while the remaining DCIS cases are associated with invasive carcinoma [1]. Pure DCIS is present in the 20% of female breast cancers [27]. This difference seems to be related to the increased awareness and regular breast screening in female patients, which leads to earlier detection and overall better prognosis.

In our case, the patient is 43 years old. He was younger than the average age of onset (67 years) as described in literature [1,4,19]. MBC seems to present from 5 to 10 years later than in women, probably due to several aspects such as a delay between onset of symptoms and timing of diagnosis (from 6 to 35 months) [19] and a general diagnosis at an advanced stage, several years after the real onset of the pathology. The late diagnosis itself could be the result of the absence of a screening programme, the lack of symptoms in the early stages and the ignorance or denial of the disease, considered as strictly feminine by men and extremely rare by the practitioners [19].

As described clearly by Foerster et al., 81.5% of male breast cancers are diagnosed at tumour stage II while 56.8% have lymph node involvement at the time of diagnosis [28].

Data from the National Institute of Surveillance, Epidemiology and End Results (SEER) show that, in United States, early forms of MBC increased from 1973 to 2012 for both men and women, but if this increase is more significant for women, men were more likely than women at stage III and IV (24.9% vs. 17.2%) [19].

The histological patterns seen in female DCIS are also seen in males but with varying frequency. Hittmair et al. [25] looked at 84 cases of pure DCIS of the male breast and found that the most common histological subtype was papillary carcinoma (74%) with a superimposed cribriform pattern. Pure DCIS cases in this series were found to be of either low or intermediate grades. To our knowledge, only a few cases of pure high-grade DCIS have been described, more recently in association with gynaecomastia and more typical risk factors [3]. There were no cases of pure high-grade DCIS in association with IP and ADH as seen in our patient.

Our diagnosis showed the importance of triple assessment or triple test. It consists of clinical assessment, radiologic assessment (mammography and/or ultrasound) and tissue biopsy (fine-needle aspiration cytology and/or core biopsy) [9]. In their review, Irwig et al. [29] showed that when any component is positive, the triple test has a sensitivity of 99.6% (95–100%) and a specificity of 62%. Similarly, the sensitivity of FNAC is consistently high (92%). The relatively low specificity can now be overwhelmed by core biopsy or other interventional procedures such as vacuum-assisted breast biopsy (VABB), performed under the guide of ultrasound, mammography or tomosynthesis (tomobiopsy). Nevertheless, the low volume of male breast makes it difficult to perform these types of procedures.

In our case, the complexity of the area in which we found both proliferative lesions and carcinoma, determined the different results of cytology and histology that, in accordance with clinical and DBT findings, suggested that we perform surgery. In fact, the presence of the asymmetry at standard 2D mammograms with the absence of a high PPV finding of the US could have been misinterpreted as a dendritic gynaecomastia. By using DBT, the identification of the architectural distortion, similar to the findings of a FBC, increased the PPV of both techniques, leading us to perform biopsy and then surgery.

The role of imaging in MBC is controversial. Because of its limited volume, the male breast is not as easy to examine with standard imaging techniques as for women, especially mammography. Furthermore, the superimposed presence of gynaecomastia could mask the lesion or other relevant findings such as architectural distortions or microcalcifications, not clearly evaluable with ultrasounds.

Physical examination is very sensitive (85–100%) for the detection of cancer but lacks specificity (PPV of 19.2%). Mammography showed the highest sensitivity (94.7%, NPV of 99.7%) and ultrasound is the most specific (95.3%) for detection of malignancy [30]. Nevertheless, if, as Evans et al. [31] suggested, the routine use of mammography could be an adjunct to physical examination in distinguishing benign and malignant processes, Adibelli et al. [32] proposed a diagnostic algorithm in which ultrasonography may be used to evaluate palpable abnormalities as the first diagnostic tool of choice. In a review of the literature, the discordance between different algorithms proposing the use of mammography [30,33,34,35,36], underlined the lack of specific data concerning the use of imaging in this specific clinical setting. In most cases diagnosis is made by triple assessment (clinical assessment, mammography or ultrasonography, and core biopsy) and ultrasound-guided core biopsy is preferred because it can enable a definitive diagnosis of invasive breast cancer [37].

**Figure 7:**
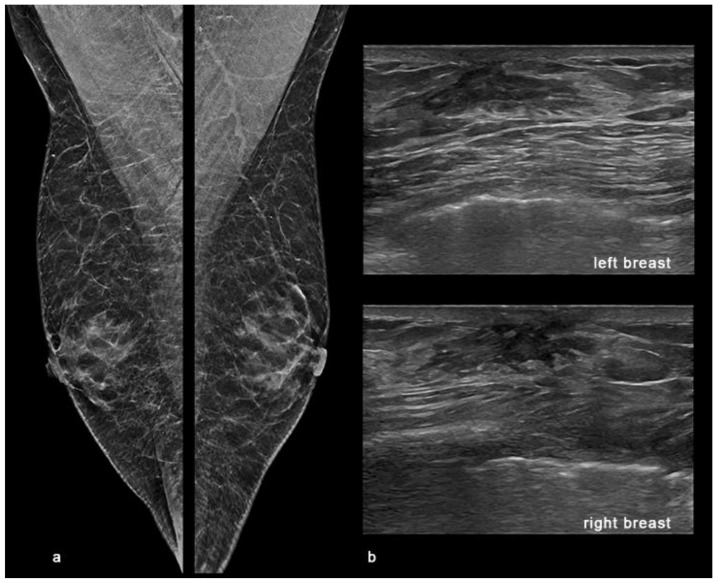
Gynaecomastia. Digital breast tomosynthesis (DBT) and ultrasound (US) showing bilateral gynaecomastia in a 74-year-old male observed at our department with tender lumps in both breasts and mastodynia at clinical examination. (**a**) The synthetic 2D reconstructed medio-lateral-oblique images (s2D-MLO) showed a symmetric increased flame-shaped sub-areolar density in both breasts. (**b**) US showed a retro-areolar, triangular, hypoechoic mass in both breasts.

The American College of Radiology Appropriateness Criteria Committee recently recommended criteria for imaging the breasts in symptomatic men [38]. The panel recommends mammography or digital breast tomosynthesis (DBT) in men aged 25 and older if there are symptoms or if physical examination suggests BC. Other studies also show the usefulness of mammography in asymptomatic men at high risk for developing BC [39]. The role of DBT in female breast cancer diagnosis is well known. The addition of DBT to standard 2D mammography reduces recall rates and improves cancer detection rates of screening mammography compared with standard 2D digital mammography alone [40,41]. DBT improves the visualization of findings that may be subtle or occult on 2D, reducing the superimposition of fibroglandular tissue, especially in dense breast and for architectural distortions [42,43,44]. In fact, the cancer detection rate of DBT in mammographically occult architectural distortions has been reported to be 21.1% [43] and 47.2% [45]. Furthermore, the substantial equivalence of standard 2D images (2DFF) with 2D synthetic reconstruction (s2D) [46], obtained after performing DBT, addresses the issues related with increasing dose in the combined use of 2DFF with DBT, allowing the use of the s2D images rather than standard 2D views. All these aspects suggested the use of DBT also in male breast imaging. Increasing conspicuousness of findings makes early detection of cancer before its progression to invasive forms possible, especially in cases like our one, in which the lesion presented as an area of architectural distortion, and in case of superimposed gynaecomastia that could mask underlying pathological findings, as happens in the case of dense breast tissue in women [47] (Figure 7).

The diagnostic impact of magnetic resonance imaging (MRI) for the male breast is not sufficiently examined. In small studies, the MRI features of benign breast diseases and breast cancers in male patients seemed to be comparable to those seen in female patients, and therefore its diagnostic use should be limited to corresponding applications [48].

To our knowledge, there are no cases of male DCIS presenting as architectural distortion described in most of the literature about MBC [23,49,50,51,52,53,54,55].

**Figure 8:**
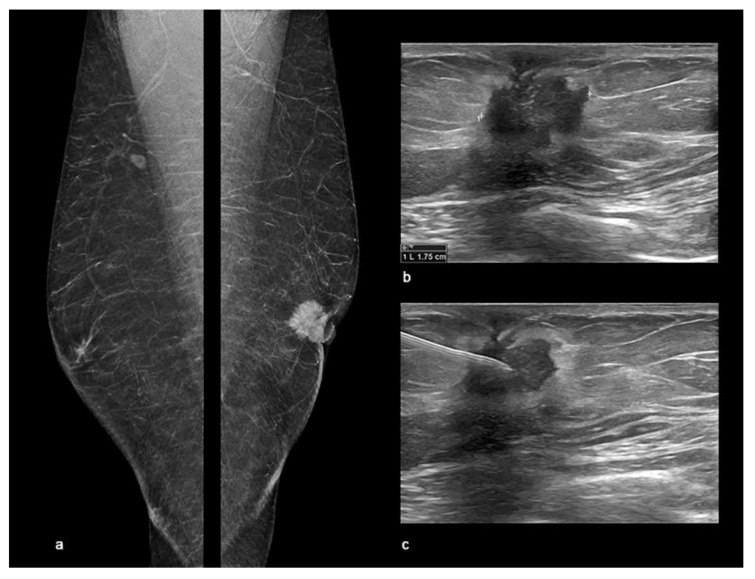
Male Breast Cancer. Digital breast tomosynthesis (DBT) and ultrasound (US) showing an invasive ductal carcinoma of the left breast in a 58-year-old male observed at our department with a palpable retro-areolar mass and nipple retraction at clinical examination. (**a**) The synthetic 2D reconstructed medio-lateral-oblique images (s2D-MLO) showed a sub-areolar dense mass with spiculated margins and skin retraction. (**b**) US showed a left retro-areolar, hypoechoic mass with indistinct border and spiculated margins of 1.75 cm. (**c**) US-guided Fine-Needle Aspiration Cytology (FNAC) resulting C5 according to the the IAC Yokohama System for Reporting Breast Fine-Needle Aspiration Biopsy Cytopathology (1st Edition, 2020).

According to this, in mammography, MBC usually occurs as a round, oval or irregular high-density sub-areolar mass with circumscribed, indistinct, speculative or micro-lobulated margins [36] (Figure 8). Microcalcifications are rare and observed in only 13% to 30% of cases [56], often with benign or nonspecific appearance [3]. In women, architectural distortion is the third most common mammographic appearance of non-palpable breast cancer, representing nearly 6% of abnormalities detected on screening mammography [56]. According to BI-RADS System [5], it was described as an appearance in which “the normal architecture of the breast is distorted with no definite mass visible”, including spiculations radiating from a point and focal retraction or distortion at the edge of the parenchyma. Chopier et al. [57] suggested classifying all the architectural distortions as BI-RADS 4, except in case of a known scar. The PPV for malignancy of an architectural distortion detected on mammography is 74.5% [58] and on diagnostic DBT, it is malignant in a third of cases. The presence of an ultrasound correlate reinforced the association with malignancy [59].

DCIS most commonly presents typically with pleomorphic, linear, or linear branching microcalcifications, but architectural distortion has been described as occurring in up to 2–10% of women. Although the most common features of DCIS on ultrasound are hypoechoic masses and microcalcifications, architectural distortion may be seen in up to 4% of cases [56]. Marino et al. [39] found that, of 163 men who underwent screening mammography, 6 were assessed BI-RADS 3 for focal asymmetry and/or architectural distortion but none of them showed malignant findings at follow-up or biopsy (dendritic gynaecomastia, post-surgical distortion). In their review, AlSharif et al. [60] found a single case of focal asymmetry that showed segmental homogenous non-mass like enhancement at magnetic resonance imaging (MRI). Instead, Isley et al. [61] described a case of male DCIS presenting as microcalcifications alone in absence of homolateral gynaecomastia.

Early diagnosis of MBC is fundamental to optimise therapeutic options and improve the survival rate. Although DCIS is extremely rare, it is an entity to be considered. Thirty to fifty percent of all male and female patients with DCIS develop invasive cancer in the following 10–20 years [60]. The American Cancer Society estimated that about 2650 new cases of invasive breast cancer will be diagnosed in the United States for 2021 and about 530 men will die (20%). For men, the lifetime risk of getting breast cancer is 1 in 833 and black people have a worse prognosis than white [62]. In Italy, the lifetime risk is 1 in 629 while the standardized incidence rates per 100,000 are 1.9, 1.5 and 1.6 for Northern, Central and Southern Italy, respectively [63]. The comparison of overall prognosis for male and female breast cancer patients is controversial. Studies reporting a poor prognosis for men have suggested that male breast anatomy may provide fewer barriers to metastasis or that more aggressive tumour biology may be the basis for survival variation [64]. However, others have found that, once separated by lymph node stage or involvement, the prognosis is the same. The reported 5-year overall survival rate for male breast carcinoma varies considerably from 40% to 80% with a specific survival rate of 89% and 72% at 5 years and 10 years respectively [19]. As most breast cancers in men are estrogen-receptor positive, tamoxifen is generally the standard adjuvant therapy [10,65,66,67,68,69]. Anti-androgen therapies may be an alternative and complementary treatment option for patients with triple-negative breast cancer with limited therapeutic possibilities [49]. Surgical decisions for a male DCIS are not easy and should be tailored. A modified nipple-sparing mastectomy could be useful in cases such as ours in which there was no involvement of the nipple-areolar complex. Sentinel node biopsy is suggested but not mandatory and it could spare axillary dissection in early stages MBC [70]. Since the disease is usually more locally advanced in men than in women, adjuvant loco-regional radiotherapy was more frequently administered to men than to women [71]. Adjuvant chemotherapy is generally considered a medium to high risk for BC. There are no prospective studies of endocrine neoadjuvant therapy and only a few reports of neoadjuvant chemotherapy [72].

Recent studies have explored the possible use of radiomics in breast disease, including distinguishing benign and malignant lesions [73], depicting the biology of tumours, differentiating between early and advanced stages, predicting cancer response to treatment and determining the risk of recurrence [74,75,76].

Radiomics is a rapidly evolving field of research concerned with the extraction of quantitative data within medical images, using advanced mathematical algorithms. Radiomic features, as they are defined, capture tissue and lesion characteristics such as heterogeneity and shape and may, alone or in combination with demographic, histologic, genomic, or proteomic data, be used for clinical problem solving [77].

Huang et al. [78] were the first to develop a model based on a radiomics analysis with traditional imaging features in mammography to distinguish male benign and malignant breast lesions. They selected five major features, including asymmetry, lesions located in non-retro-areola region, lesion eccentricity, skin thickening and calcification to differentiate MBC. Finally, they developed and validated an imaging-radiomics nomogram for clinicians to determine breast cancer risk for every male patient, recommending biopsy or surgery for all patients at high risk. In our case, there was at least three features suggesting cancer (asymmetry, eccentricity, and non-retro-areola region) and we think that radiomics could have been and will be a useful tool to help in the diagnosis of MBC even if further studies are necessary to validate its use.

## 4. Conclusions

The understanding of biology, clinical presentation and imaging features, genetics, and management of MBC is evolving but there still remains a large knowledge gap due to the rarity of this disease. Furthermore, there are no standardized protocols for male breast imaging and radiologists are generally less familiar with breast disease in male patients than female patients. Therefore, the knowledge of clinical and imaging presentation of DCIS is fundamental for the radiologists and clinicians involved.

Our case report is a unique mammographic and clinical presentation of a pure high-grade DCIS in a young male, associated with IP and ADH, without gynaecomastia or significant risk factors.

Atypical ductal hyperplasia (ADH) significantly increases the risk of breast cancer in women. However, little is known about the implications of ADH in men. In their study, Coopey et al. [11] showed no evolution to BC and this suggested that ADH in men does not pose the same risk as ADH in women.

Furthermore, our patient showed a clear, not bloody, nipple discharge, probably related to the focal ductal ectasia with the papilloma. This experience confirmed that a nipple discharge of any kind should be always considered suspicious for BC [79] and it could be the only clinical sign of carcinoma in situ.

**Figure 9:**
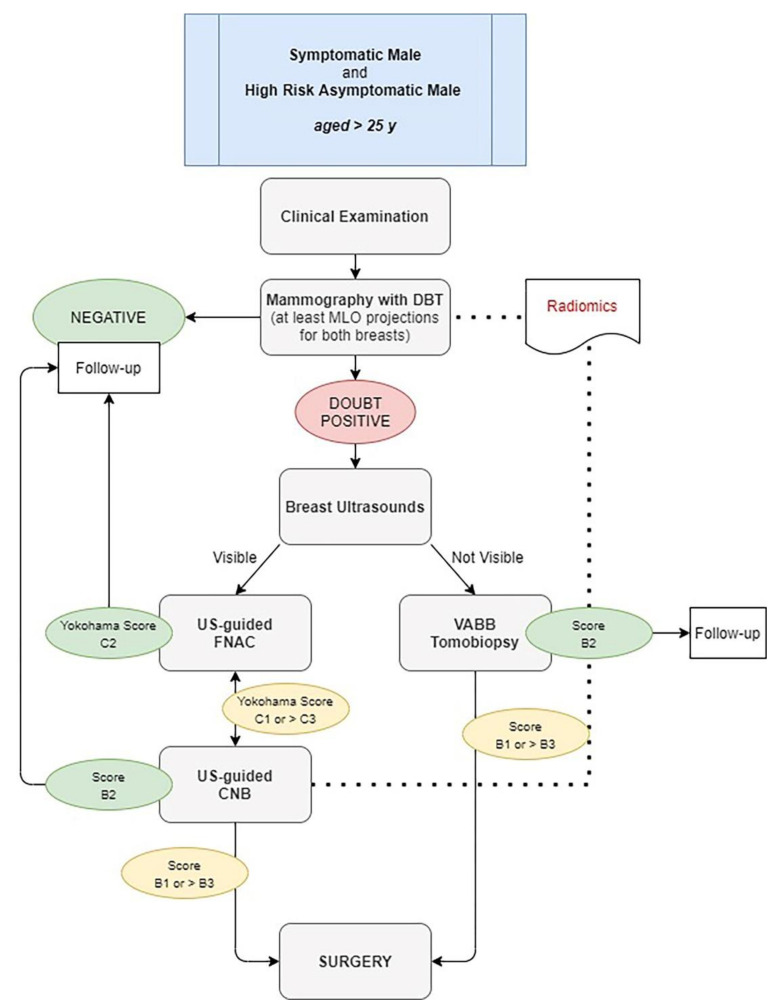
Diagnostic Algorithm for both symptomatic and high-risk asymptomatic male aged > 25 years. DBT: digital breast tomosynthesis. US: ultrasound. VABB: vacuum-assisted breast biopsy. FNAC: fine-needle aspiration cytology. CNB: core-needle biopsy.

In conclusion, although knowledge about FBC can inform MBC diagnosis and treatment, molecular and clinic-pathologic features differ between two genres. There is an unmet need for research and diagnostic-therapeutic options for this disease. Since mammography and ultrasonography are useful to diagnose a carcinoma in initial stages, avoiding unnecessary biopsies, we propose a single simple algorithm to help radiologists who interface male breast disease in both symptomatic and asymptomatic patients at high risk elder than 25 years old. In our diagram (Figure 9), we suggest to perform physical examination with at least a single mammography MLO projection for both breast, better if in digital breast tomosynthesis (DBT) with synthetic 2D reconstruction (s2D). In case of doubtful findings on both clinical and imaging first examination, other mammographic views could be performed (cranio-caudal, medio-lateral, magnification or spot-views).

Ultrasonography could be an adjunct to mammography, especially to perform US-guided cytology. This type of triple test could avoid unnecessary biopsy and surgical procedures. Therefore, in case of suspicious findings (BI-RADS > 3 or IAC Yokohama System > C3), a core or vacuum-assisted breast biopsy should be performed before surgery.

However, since the radiologists’ experience affected the process of discriminating benign and malignant male breast lesions, the potential support of radiomics could enhance clinical decision-making. In fact, imaging features are often assessed visually and described qualitatively. This could lead to a large variability while it would certainly be more appropriate to objectively and reproducibly quantify these aspects. The use of a standardized imaging-radiomics nomogram, based on the evaluation of well-determined features, could help radiologists to determine breast cancer risk for every male patient, recommending biopsy or surgery when at high risk.

Evidence regarding the cost-effectiveness of healthcare technology is increasingly required to inform the decision on whether to fund and implement new treatment or diagnostic tools [80]. Many decision-making bodies require interventions to be assessed using cost per quality-adjusted-life-years (QALYs) [81], a single summary measure combining life expectancy with individuals’ relative preferences for health states in terms of quality of life [82]. Nevertheless, evaluating the true cost-effectiveness of breast cancer prevention will remain problematic [83]. Female breast cancer screening is affected by many challenges related to the assessment of its harms and benefits, such as anxiety or reassurance, the long-term implications of breast surgery, the improvement of therapies versus the advantages of early detection, over diagnosis versus undervaluation. Male breast cancer is a rare pathology but it could be an interesting field of research for both clinical and economical aspects. To analyse the costs of the diagnostic-therapeutic care pathway for each patient, using the micro-costing “bottom-up” approach [84], could be useful to gather all the needed information to assess better qualitative and quantitative performance in according to the best practice in clinical governance.

Our protocol could be an example of a male breast cancer prevention model, optimizing the use of resources in according to the criteria of “3Es”: efficiency, effectiveness and economy.

## Figures and Tables

**Table 1:** Genetic Analysis. Panel of genes.

MATERIALS	Genomic DNA from Venipuncture
PANEL OF GENES	ATM, APC, BARD1, BRCA1, BRCA2, BRIP1, CDH1, CHEK2, EPCAM, FAM175A, MLH1, MRE11A, MSH2, MSH6, MUTYH, NBN, PALB2, PIK3CA, PMS2, PMS2CL, PTEN, RAD50, RAD51C, RAD51D, STK11, TP53, XRCC2.
RESULTS	Molecular analysis performed did not identify pathogenic variants of sequence and rearrangements in the investigated genes.
COMMENTS	The analysis did not reveal the presence of clinical-relevant genetic alterations of the genes investigated.
METHODS	Method 1: Next Generation Sequencing (NGS) and CNV analysis (MiSeq/NextSeq, Illumina), made with the Hereditary Cancer Solution kit (HCS, Sophia Genetics), of the coding exons and of the adjacent intronic sequences (+/−25 bp) of the investigated genes. Minimal coverage per locus: 100×. Data analysis made with software DDM (current version, Sophia Genetics). Method 2 (confirmation): Sanger sequencing of interested region; analysis of electropherogram with Sequencer 5.4 (Gene Codes Corporation, Ann Arbor, Michigan). Sensitivity and specificity > 99%. Reference Genome: GRCh37/hg19. Numeration starting from ATG (A = +1)
NOTE	The nomenclature used for the description of the variant is in accordance with the current Human Genome Variation Society (HGVS) guidelines (v20.05), May 2020. The interpretation of the clinical significance is in accordance with the disease database of reference (ClinVar) and “Standards and guidelines for the interpretation of sequence variants: a joint consensus recommendation of the American College of Medical Genetics and Genomics and the Association for Molecular Pathology”. Richards et al., 2015. Benign variants (c1) are not listed in the report. The method does not identify tissue mosaicism.

## Data Availability

The reported data come from saniarp.it, the Caserta LHA reporting database and from the register of our daily activities.
